# An Efficient Pest Detection Framework with a Medium-Scale Benchmark to Increase the Agricultural Productivity

**DOI:** 10.3390/s22249749

**Published:** 2022-12-12

**Authors:** Suliman Aladhadh, Shabana Habib, Muhammad Islam, Mohammed Aloraini, Mohammed Aladhadh, Hazim Saleh Al-Rawashdeh

**Affiliations:** 1 Department of Information Technology, College of Computer, Qassim University, Buraydah 51452, Saudi Arabia; 2 Department of Electrical Engineering, College of Engineering and Information Technology, Onaizah Colleges, Onaizah 56447, Saudi Arabia; 3 Department of Electrical Engineering, College of Engineering, Qassim University, Unaizah 56452, Saudi Arabia; 4 Department of Food Science and Human Nutrition, College of Agriculture and Veterinary Medicine, Qassim University, Buraydah 51452, Saudi Arabia; 5 Department of Cyber Security, College of Engineering and Information Technology, Onaizah Colleges, Onaizah 56447, Saudi Arabia

**Keywords:** artificial intelligence, crop diseases, convolutional neural network, Faster-RCNN, machine learning, object detection, pest detection, pattern recognition, YOLOv5

## Abstract

Insect pests and crop diseases are considered the major problems for agricultural production, due to the severity and extent of their occurrence causing significant crop losses. To increase agricultural production, it is significant to protect the crop from harmful pests which is possible via soft computing techniques. The soft computing techniques are based on traditional machine and deep learning-based approaches. However, in the traditional methods, the selection of manual feature extraction mechanisms is ineffective, inefficient, and time-consuming, while deep learning techniques are computationally expensive and require a large amount of training data. In this paper, we propose an efficient pest detection method that accurately localized the pests and classify them according to their desired class label. In the proposed work, we modify the YOLOv5s model in several ways such as extending the cross stage partial network (CSP) module, improving the select kernel (SK) in the attention module, and modifying the multiscale feature extraction mechanism, which plays a significant role in the detection and classification of small and large sizes of pest in an image. To validate the model performance, we develop a medium-scale pest detection dataset that includes the five most harmful pests for agriculture products that are ants, grasshopper, palm weevils, shield bugs, and wasps. To check the model’s effectiveness, we compare the results of the proposed model with several variations of the YOLOv5 model, where the proposed model achieved the best results in the experiments. Thus, the proposed model has the potential to be applied in real-world applications and further motivate research on pest detection to increase agriculture production.

## 1. Introduction

In agricultural production, pest detection has always been a serious problem, which is responsible for 20% of annual crop losses globally [1,2]. In 2021, the affected areas caused by pests and major diseases in China had reached almost 400 million hectares. Therefore, timely detection of crop diseases and pests is crucial to agricultural production, which has a significant impact on grain production, agricultural development, and farmers’ income increase [3]. Building an artificially intelligent model based on agricultural image processing is one of the more effective ways to detect pests and classify them according to the class label [4], which can give an efficient response and intervention to agricultural production, increase the effectiveness of pest detection model, reduce the losses in the agricultural production.

Therefore, researchers used traditional machine learning (ML) [5,6], and deep learning-based models for an efficient pest detection system. The traditional methods of detecting insect pests based on morphological features are limited by the need for trained taxonomists to make accurate identifications [7]. It is important to note that traditional methods for pest detection come with many limitations. Recently, several methods for automatic pest detection using traditional machine learning are proposed [8]. For example, Faithpraise et al. [9], proposed K-means clustering algorithms for pests detection. Manual feature extraction and relative filter were used to recognize various species of pests, which is time-consuming when the dataset is large. Rumpf et al. [10], proposed a support vector machine and spectral vegetation-based sugar beet diseases recognition. These methods are capable of pest detection; however, several limitations restrict the traditional ML-based model from real-world implementation such as when multiple features have to be extracted manually, traditional ML algorithms are often inefficient. Secondly, in the traditional ML-based methods, the manual features extraction and classification is time-consuming, tedious, error-prone and requires computer experts. 

The concept of deep learning refers to ML that uses multilevel neural networks for learning and extracting automatic end-to-end deep features [11,12,13,14,15,16,17]. This strategy improves detection performance while reducing the time and effort of manual feature extraction [18,19,20]. A DL-based method for pests and disease recognition in tomato leaves was proposed by Shijie et al. [21]., which obtained an average accuracy of 89%. However, this approach is only applicable to recognize pests in a simple background, which restrict the system from real-world implementation. Gandi et al. [22]., employed a Generative Adversarial Network to augment the dataset, and then the augmented dataset was fed to a pre-trained CNN model for plant disease classification. Another approach, used a DL-based method for fruit fly identification and obtained 95.68% accuracy [23]. DL-based methods to recognize ten different pest species were proposed by Dawei et al. [24]., and achieved 93.84% accuracy. Analysis of previous work shows that DL methods significantly improve pest classification performance. However, several factors hinder the existing of DL-based methods from real-world implementation such as lack of suitability for mobile devices, deficiency of robustness, lower accuracy, and a high equipment cost. Therefore, we propose a novel method for efficient pest detection based on a modified YOLOv5 model. The YOLOv5 model has several advantages over state-of-the-art object detection model, such as fast inference speed, higher mean average precision (mAP), strong customization, and lower computational complexity, which ensures the detection accuracy. We further improve the YOLOv5 model with several modifications to achieve higher mAP with lower computational cost. In the designing of the proposed model, we firstly modified the cross stage partial network (CSP) to focus more on the shallow feature extraction while the feature extraction module iterates to get more details. Furthermore, the modified select kernel (SK) in the attention module has been proposed in residual blocks, where the channel dimension is reweighted and fused. In the detection head, the multi-scale features detection is improved to detect weak and small objects. To the end of this, the major contribution of the proposed work is as follows:

1. We develop a medium-scale pest (insects) detection dataset that includes diverse images, captured in a challenging environment, where the object has high visual similarity with the background. The dataset consists of five different classes that allow a network to efficiently detect and recognize the pest species.
2. We propose YOLOv5s models with several modifications such as extending the CSP module, improving the SK Attention module, and modifying the multiscale feature extraction mechanism to efficiently detect pest, and reduce computational cost.
3. We perform experiments with various versions of YOLOv5 using a self-created dataset, where the proposed model achieves the best results in terms of model accuracy and time complexity analysis.

## 2. Related Work

The agriculture field plays a vital role to hike the economy of the countries, so it is essential to find-out harmful pests in natural environments. Therefore, several researchers have been working to develop an automatic system [25], for the recognition of insects in the agriculture field. Cheeti et al. [26], utilized a Convolution Neural Network (CNN) [27] and You Look Only Once (YOLO) model to classify and detect pests in the agriculture fields. They used their own manually created dataset from the internet through which they obtained promising performance in terms of the testing results. As follow-up research by Mique et al. [28], developed a technique for detecting rice pests and diseases with the help of CNN [29], and image processing. They trained their model on collected images from the internet and achieved 90.9% training results in terms of accuracy. In addition, they deployed their proposed model on the mobile application for public use. However, their technique is quite expensive and needs further improvement in terms of accuracy. Nam et al. [30], proposed a pre-trained Single Shot MultiBox Detector (SSD) model with some fine-tuning strategies for the accurate detection and classification of trap insects. They employed Deep CNN (DCNN) [31], and achieved 84% and 86% of testing accuracies using the custom dataset. However, their proposed model had an enormous performance as compared to the state-of-the-art. Li et al. [32], employed the DCNN networks (namely Faster-RCNN, Mask-RCNN, and Yolov5) for the effective detection of insects in agriculture fields using the IPI02 dataset. They achieved a promising performance and stated that the Faster-RCNN and Mask-RCNN have better results than Yolov5, which leads to 99% while Yolov5 has 97% performance in terms of accuracy, but the real-time testing speed for pest detection and localization of Yolov5 was faster than the Faster-RCNN and Mask-RCNN. Alsanea et al. [33], proposed an effective region-based CNN to detect and classify red palm weevil (RPW), their model achieved optimal performance in terms of evaluation matrices using the RPW dataset, however, the inferencing speed and model complexity restricted their method from a real-time implementation. Kouba et al. [34], utilized a sensor-based technique for monitoring agriculture. They used a customs dataset, which was created with the help of accelerometer sensors. In addition, their method is deployed on a mobile application for public use, which early detects the RPW based on their movement. Hu et al. [35], used a near-infrared imaging technology-based method and YOLOv5 for the accurate classification and detection of the pest in the agriculture fields. They obtained promising performance which was 99.7% of mAP using their custom dataset. Burhan et al. [36], compared the performance of four pre-trained deep learning models (namely VGG16, VGG19, ResNet50, and ResNet50V2) with some fine-tuning strategies for the detection of pests and identification of rice field diseases in agriculture fields. They achieved comparatively promising performance with an accuracy of 86.799%. However, their proposed model needs further improvement to enhance the performance in terms of evaluation matrices. Svenning et al., [37], proposed a pretrained CNN-based model with some fine tuning techniques for the classification of carabid beetle species. They achieved an average classification of 74.6% while it classified 51.9% of test images accurately to species level. The speed of their proposed model in the testing phase bounded the model from a real-time implementation. Chen et al. [38], proposed an AI mobile-based model for the detection of pests in the agriculture fields using a custom dataset. They focused on different types of pretrained deep learning (DL) models named faster region-based convolutional neural networks (R-CNNs), single-shot detectors (SSDs), and YOLOv4 for correct identification. They stated that the YOLOv4 achieved comparatively better performance in terms of F1-score, i.e., 100% f1-score in mealybugs, 89% in Coccide, and 97% in Diaspididae. 

Liu et al. [39], proposed an end-to-end region-based DL model named PostNet for multi-class classification and identification of pests using the MPD2018 database. Their article is focused on three tiers of processing, in the first tier, they utilized the concept of Channel-Spatial Attention (CSA) to enhance the performance of the model. The second tier is based on region proposal network while the third is focused on the replacement of the fully connected (FC) layers. The experimental results show that the PestNet achieved 75.46% mAP, which is comparatively better than state-of-the-art methods. However, their model has enormous performance and needs more enhancement in terms of evaluation matrices. As a follow of research Liu et al. [40], implemented a DL-based YOLOv3 model to detect pest and tomato diseases in a natural environment in the agriculture fields using a custom dataset. Similarly, Legaspi et al. [41], implemented a DL-based YOLOv3 model for the classification and detection of pests especially whiteflies and fruit flies using a custom dataset. Their research is focused on hardware implementation namely Raspberry Pi, desktop, and web applications for public use. Their experimental result demonstrates that the model obtained 83.07% performance in terms of accuracy for the classification and detection of pests. However, their technique needs further enhancement for accurate prediction. Lim et al. [42], proposed a pre-trained convolution-based AlexNet model with several fine-tuning strategy techniques for the accurate classification of insects in real environments. Karar et al. [43], presented a DL technique based on the mobile application to recognize pests in the agriculture fields. Their experiment results are focused on Faster-RCNN for the accurate identification of pests in a real environment. The experiments of the proposed model show that the model achieved 99.0% accuracy in the testing phase. Likewise, their proposed model is comparatively better than other state-of-the-art DL architectures such as SSD and traditional back propagation (BP) neural networks. G.M.Esgario et al. [44], proposed a CNN model for automatic biotic stress detection in coffee leaves. They also developed a mobile app to assist coffee farmers. Furthermore, Habib et al. [45] proposed a traditional machine learning-based model to automatically recognize and classify the brown- and yellow-rusted diseases in wheat crops. In [46], the researchers proposed a novel DeepPestNet model for pest recognition, which consists of eight convolutional layers and three fully connected layers, and achieved higher performance. However, their method is computationally expensive. 

Based on the literature review, traditional machine methods learning need two basic steps such as features extraction and classification, where the selection of an effective features extractor and classifier is a major concern, time-consuming, and requires experts in the fields. The deep learning model leverages these issues by adopting an end-to-end features extraction mechanism, however, in the literature some of the methods produced limited results and the other requires large computational resources, which cannot be deployable over resource constraints devices for real-time pest recognition.

To cope with this, we propose a DL-based YOLOv5 pipeline for the real-time recognition of pests in the natural environment. We conduct our experiment on a custom dataset and achieved promising performance in terms of accuracy. In addition, our proposed model is also focused on reducing the false prediction rate and outperforming other state-of-the-art methods. In the coming section, we provide details information on the proposed method of our article. 

## 3. The Proposed Method

**Figure 1:**
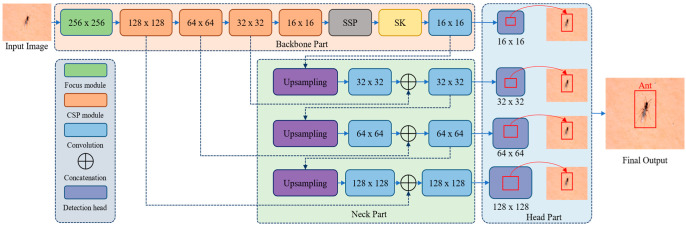
Illustrated the overall framework of the proposed model for pest detection.

In this section, we provide a detailed description of the proposed DL-based object detection model. For object detection, one-stage DCNN such as YOLOv3, YOLOv4, and YOLOv5 have obtained remarkable performance in terms of inference speed, model size, and accuracy. In these versions, the YOLOv5 is the most recent version, which utilizes various network structures and two different varieties of CSP modules to increase the YOLOv4 performance. Therefore, this work presents a method based on YOLOv5 for pest detection and recognition by modifying the network structure. As a result, the proposed model obtained good performance to detect weak and smaller objects efficiently and effectively in the tested images. The proposed framework is demonstrated in Figure 1, which consists of three parts that are backbone, neck, and head. The dimension of the input images changes from 512 × 512 × 3 to 256 × 256 × 4 after the focus module operation. Then, we use an extended CSP module in the backbone to extract promising features from shallow and deep feature maps after the operation of the focus module. We also introduce an attention mechanism in the CSP, which focuses more on small objects in an image. In the SPP module, a concatenation operation is used for fusing the acquired results from channel dimension using four pooling layers that efficiently solve the problem in anchors and feature maps. In the neck part, a path aggregation network (PANet) is used for feature pyramid generation and the bottom-up and top-down approach is used for multiscale features fusion obtained from the backbone network and increase the object detection performance using different scales. Finally, in the head part, we use four different sets of feature maps for object detection at different scales with recognition of class labels and score predictions.

### 3.1. Extended CSP Module

**Figure 2:**
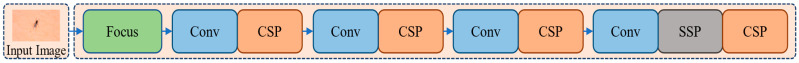
The Backbone part of the proposed work with the extended CSP module.

In the DL-based model the hidden layers are gradually increasing, which extracts the semantic information of high-level features more precisely, with a reduced number of dimensions. In contrast, the dimensionality of the shallow layer is higher, which extracts the low-level feature in the network. For weak and small object detection with a few features, deep CNN may fail to achieve promising results. To increase the feature extraction capability for the weak and smaller objects in the input image, it is essential to make full use of the high-level features of the CNN in the shallow layer. Therefore, in the features extraction part, we extended the thickness of the CSP module in the shallow layer for feature extraction. This strategy is a follow-up in the upcoming layer to extract the multi-scale object features from shallow to deeper layers. Furthermore, when deepening the CSP in the entire network for feature extraction by controlling the depth and width factors, we expand the CSP module thickness to extract shallow features. The backbone part of the proposed model is shown in Figure 2. This strategy increases the model size and complexity but also increases the capability to extract prominent features from the shallow layers which are beneficial to detect small and weak objects in images. Moreover, the CSP part splits the features maps into two branches for feature extraction and then fuses them, which can obtain a richer gradient combination with a reduced number of calculations.

**Figure 3:**
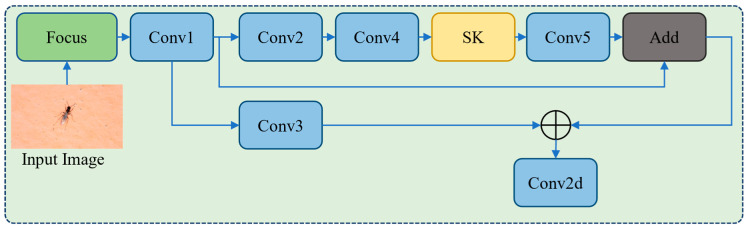
The extended CSP module of the proposed work with modified SK attention module.

In the proposed model, we stack the convolution and CSP modules three times after the focus module. In this regard, the shallow layer is expended to the similar size of CSP module feedback as the deeper layer, and the feature maps with different sizes are acquired step-wise. Then, we obtain full fine-grained features of shallow and deep semantic information as shown in Figure 3, where convolution represents three basic operations that are 2D convolution, batch normalization, and activation function. By the concatenation operation, the feature maps that have two convolution branches and the attention (SK Layer) are fused. Finally, a 128 × 128 features vector is extracted in the shallow layer. When comparing the proposed model with the YOLOv5m model that added 108 layers to extend the shallow layer of the CSP module, we only added 18 layers to the network without compromising the network performance.

### 3.2. Modified SK Attention Module

**Figure 4:**
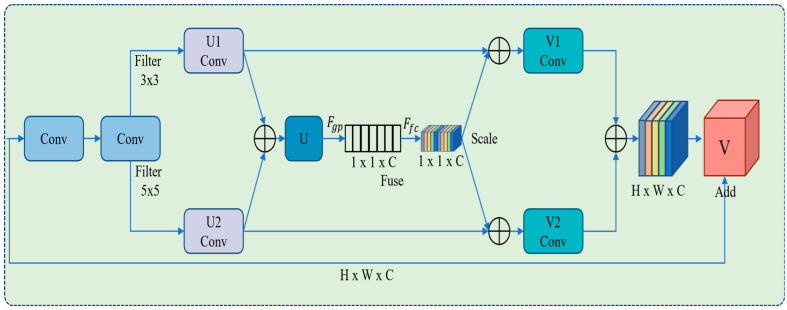
Represents the internal architecture of the modified SK attention module of the proposed work.

The visualization part of any system focuses on a piece of information that helps to evaluate the image and ignores unnecessary information. In the DL-based model, an attention module can be used in the residual blocks of the shallow layer in the feature extraction stage for prominent feature selection and assigned more weights to weak and small objects to enhance feature extraction capabilities for accurate object recognition/detection [47,48]. The SK Net model adaptively modifies the receptive field size according to the multi-scales of the input information. Thus, we propose an improved version of the SK attention mechanism in each CSP and utilizes two convolutional operations with different filter sizes for channel weight learning. The output feature maps continue to perform 1 × 1 convolution as shown in Figure 4. 

The improved version of the SK attention mechanism is directly employed in the residual blocks, which is divided into three parts such as (1) Split: separates the input vector to perform convolution operation with two different filters size i.e., 3 × 3 and 5 × 5 to achieve the output vectors $U_{1}$ and $U_{2}$ and acquired U after the addition process. (2) Fuse: utilizes the global average pooling denoted by ($F_{gp}$) for the matrix compression to 1 × 1 × C, and employs a channel descriptor for the representation of each channel information. Thus, the dependency between channels is determined, which is mathematically formulated in Equations (1) and (2). The fully connected layers ($F_{fc}$) established the relationship between the channels flexibility and nonlinearity. In the proposed work, we use two $F_{fc}$ layers to add nonlinearity, fit the complex correlation between channels, decrease the training parameters and computations as much as possible, and obtain the weight value, as given in the following Equation:

(1) $$F_{gp}\left(U\right)=\frac{1}{W\times H}\overset{W}{\underset{i=1}{\sum }}\overset{H}{\underset{j-1}{\sum }}U\left(i,j\right)$$

where *W* represents the weight, *H* height, *i i*th row, and *j j*th column of the given image, respectively.

(2) $$F_{fc}\;\left(F_{gp},\;\varpi \right)=\delta \left(\beta \left(F_{gp},\;\varpi \right)\right)$$

The ϖ is used for weight, δ is the Relu activation, and β is the batch normalization. 

(3) Scale: a simple weighting operation, where the calculated weight values in the fusion stage are multiplied with the original matrix to achieve the outcome of the SK blocks. This strategy improves the feature extraction process for weak and small object detection. Then the matrix columns are fused to utilize the shallow and deep layer features. We use a fully connected layer, where a sigmoid function is directly multiplied by the vector *U* to achieve the vector *V* as given in Equation (3).

(3) $$F_{scale}\;\left(U,\;F_{fc}\right)=V1+V2=U_{1}*F_{fc}+U_{2}*F_{fc}$$

The $F_{scale}\;\left(U,F_{fc}\right)$ is channel-wise multiplication to multiply the feature maps of *U* with the obtained weight of $F_{fc}$ stage, and outputting the weighted feature. In the proposed model SK is an efficient and effective module that can be directly used in the network. The SK module has a strong model generalization capability by obtaining different receptive field features and an adaptive adjustment structure that significantly detect and recognize small and even large pest in the tested images. 

### 3.3. Multiscale Feature Detection

In the YOLOv5 object detection model, three kinds of output feature maps are used for object detection with different sizes, which utilizes 8 different downsampling output features maps for small object detection. The object in the proposed dataset for pest detection and recognition is weak and small. Therefore, we employ a feature scale to focus more on the smaller objects. When the feature maps are upsampled to 64 × 64 size, we continue to upsample the feature maps to acquire 4 downsampling feature maps. In the meantime, the extended 128 × 128 feature maps are combined with the similar size feature maps of the second layer in the backbone part of the network to make full use of the deep and shallow layer features. After the fusion of multi-scale features, the four different scales of these features are 18 × 18, 32 × 32, 64 × 64, and 128 × 128. The YOLOv5 adaptively computes suitable anchors according to different datasets, which makes the convergence capability of the model simple and detects the object with different scales. The first step is the input image selection, which will be used for the prediction. In the second step, we use four different layers for detection i.e., P2, P3, P4, and P5, which predict the values of central point tx, ty, the height th, width tw, and the confidence score. Afterward, a loss function between the ground truth and the model prediction is calculated for each detection layer. Through this, our model gradually optimizes the error rate and increases the generalization capability. The loss function for each detection layer is similar, which is obtained by computing the sum of the class loss, confidence loss, and bounding box regression loss. The mathematical expression of the P2 detection layer loss is given in Equation (4).

(4) $$loss_{p2}=loss_{class}+loss_{object}+loss_{boundin\_box}$$

where the bounding box loss utilizes CIoU, the class loss is computed according to the categorical cross entropy, and the confidence loss is obtained by categorical cross entropy with logistics loss to fit numerical stability. 

### 3.4. Psuedo Code Algorithms

According to our model structure, the pseudocode is designed, which explains the training procedure of our model step-by-step as presented in Algorithm 1.
**Algorithm 1: Psuedocode of the proposed model****Input:** Dataset samples S = {(X_1_, Y_1_),(X_2_, Y_2_),…,(X_n_, Y_n_)}. The S is categorized into a training set (Train_X_, Train_Y_), a validation Set (val_x_, Val_y_), and a testing set (test_x_, test_y_), where x is the number of pest images, and y is the corresponding image labels. T denoted the number of training epochs. **Output:** converge modelLoad the (TrainX, TrainY), and (valx, Valy);Augment the (TrainX, TrainY);**Begin:**Initialize weights and biases.**For** m = 1, 2, 3, …, T:Features extraction using CSPInput the feature SK Attention ModuleGenerate the attention map using SK Attention ModuleFed the extraction features from SK Attention Module to Multiscale Feature Detection Weight the multiscale feature maps, and calculate the output of the Multiscale Feature Detection.Model fit (Optimizer, (TrainX, TrainY)) → (M(m))Model evaluate (M(m), (ValX, ValY)) → mAP(m).**End For**Save the optimal model which has max mAP in T epochs.**End**Load the testing set;Load the optimal model in terms of object detection performances.

## 4. Dataset Collection

Dataset collection is a major part of model training in the field of artificial intelligence. In this article, we develop a new dataset that includes diverse images, captured in a challenging environment, which consists of five different classes: ants, grasshoppers, palm_weevil, shield_bug, and wasps. The ants class includes 392 frames, the grasshopper class has 315 images, palm_weevil class has 148 images, shield_bug class has 392 images, and wasps class has 318 images as tabulated in Table 1. 

We annotate the dataset according to the object detection model using labeling tool which is publicly available on GitHub (accessed on 23 June 2022). The labeling is written in Python programing language and uses Qt cross-platform Graphical User Interface (GUI) toolkit (Qt GUI 5.15.11). The YOLO-based model required .txt annotated file for data labeling, therefore we annotate the dataset according to the YOLO formats. 

**Figure 5:**
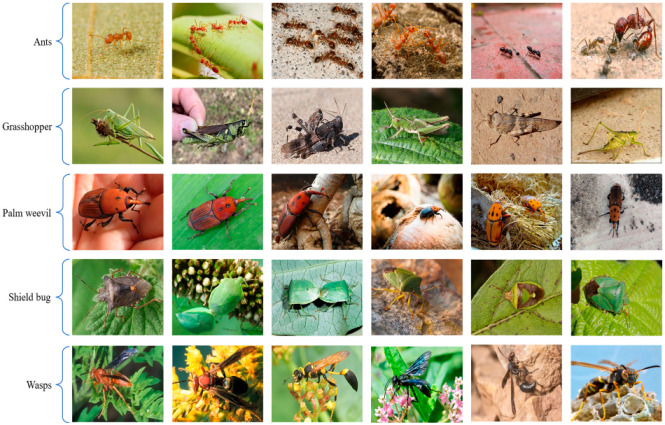
Sample images of each class of the self-created dataset for early pest detection.

All images have the same size, i.e., 512 × 512 and represent the three channels (Red, Green, and Blue). In addition, the datasets are separated into three portions thar are training, validation, and testing, where the training sets consist of 70% data, a validation sets consist of 20%, and a testing sets consist of 10%. Likewise, we implement a pre-trained YOLOv5s-based model with some fine-tuning strategies using the proposed dataset for the detection of pests in the agriculture fields. Our proposed model achieves promising performance in terms of evaluation matrices as compared to state-of-the-art methods. Sample images of each class of the dataset is presented in Figure 5.

## 5. Experiments and Results

This section provides detailed information about the dataset and the implementation of the pre-trained YOLOv5 architecture. In Section 5.1, we discuss the experimental setup of the paper; followed by the convergence of the proposed model, and then the experimental evaluations are presented.

### 5.1. Experimental Setup 

The experimental results of this article for insect detection are conducted in Pytorch with CUDA support. All the experiments were performed on the windows operating system, equipped with a Core i7-9700KF CPU, and graphics NVIDIA Corporation TU104 (GeForce RTX 3070 Super GPU) with 8 GB of RAM. To assess the testing performance of the YOLOv5 architecture, it is difficult to use the existing evaluation metrics for each problem. The precision, recall, and mAP are utilized in our experiments as the evaluation matrices and the detailed description of these metrics are available in [49,50,51,52]. 

Precision is a measurement matric that is based on a confusion matrix, used to check model performance in the field of machine learning and deep learning. In addition, it is referred to as true positive samples divided by true positive and false positive samples, as formulated in Equation (5).

(5) $$P=\frac{TP}{TP+FP}$$

Recall sometimes referred to as ‘sensitivity’ [53]. It is only dependent on the positive samples of the data and does not care about negative samples. In addition, it is calculated as the number of true positive samples divided by the number of true positive and false negative samples, as given in Equation (6).

(6) $$R=\frac{TP}{TP+FN}$$

Mean average precision (mAP) is used to evaluate the object detection models, which measures the ground-truth bounding box to the localized box and gives a score. The highest score leads the model toward accurate detection, as formulated in Equation (7).

(7) $$mAP=\frac{1}{N}\overset{N}{\underset{i-1}{\sum }}AP_{i}$$

whereas TP describes the number of positive detected samples, FP indicates the number of negative detected samples, and FN represents the number of positive samples that are not accurately classified. 

### 5.2. Convergence Results of the Proposed Model

**Figure 6:**
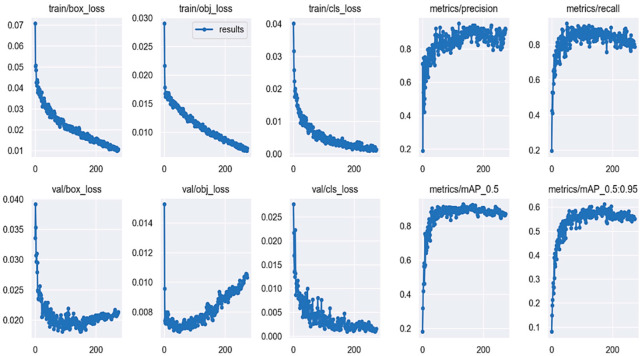
Illustrates the model effectiveness using different evaluation metrics, the X-axis represents the number of epochs and Y-axis are the corresponding score of each evaluation matrix.

First, we extract the images from open-source directories and a manual labeling technique is utilized to label each pest by its name to train the YOLOv5-based architectures for detection to protect the agriculture fields. In the experiments, we use 200 epochs with a batch size of 10 to train the model. To obtain better performance, the Stochastic Gradient Descent (SGD) algorithm is utilized to train the model better and optimize the network during training. Furthermore, we store the optimal trained weights of the model after completing the training process. The result of the model is evaluated using the validation and test images. In this paper, the training and validation sets are fed to the model as input for training. The loss graphs of the training and validation is determined after 200 epochs as mentioned in Figure 6, which contains detection frame, object loss, classification loss, precision, recall, and mean average precision (mAP). 

The loss graph represents the model performance that how our model is accurately predict the object. The model can achieve the target task when the loss function reaches a smaller value. The object loss function is focused on measuring the probability of the target task based on the area of interest. Higher accuracy depends on the smaller value of the loss function. The classification loss function can accurately classify the object category. The accurate classification of the object depends upon the lower loss as shown in Figure 6. Similarly, precision and recall are the model performance measuring terms in ML and DL. The higher precision and recall are the evidence of model accuracy as presented in Figure 6.

In Figure 6, the value of the loss function leads to declining during training, and the model parameters and their weights are continuously updated based on the SGD algorithm. After a few epochs, the model is capable of constantly reducing the loss function value while it is able to rapidly enhance the accuracy, recall rate, and precision as shown in Figure 6. Our proposed model achieves optimal performance in terms of evaluation matrices such as precision, recall, mAP, detection frame, object loss, and classification loss. The loss function value of the model in training and validation sets leads approximately to downward trends while the precision, recall, and mAP were on the peak on 200 epochs. 

**Figure 7:**
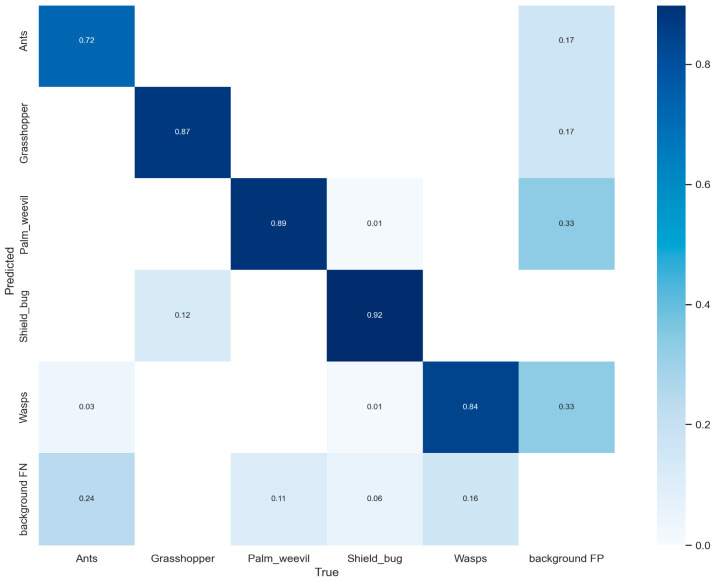
Represents the confusion matrix of the proposed model using a self-created dataset.

The performance of the proposed model is further investigated by the confusion matrix using the test dataset as shown in Figure 7. In the confusion matrix, we have five different categories of pests including one extra category named background FN. The basic purpose of the background FN category is to highlight the none detected object in the image. To further investigate the confusion matrix of our model, the correct prediction of ants, grasshoppers, palm-weevil, shield_bug, and wasps’ classes is 0.72, 0.87, 0.89, 0.92, and 0.84, respectively. 

### 5.3. Comparing the Proposed Model with the Various Versions of YOLOv5 Models and the Current State-of-the-Art Models

In this section, we compare the performance of our model with nine different versions of state-of-the-art models. The performance of the proposed model is comparatively higher than the other models for pest detection such as ants, grasshoppers, palm-weevil, shield-bug, and wasps as described in Table 2. In the experiments, the Faster-RCNN achieves the second best performance as compared to other models. However, our model surpasses the Faster-RCNN by achieving higher values of precision, recall, and mAP, i.e., 0.018, 0.015, and 0.011, respectively. During the experiments, the lower performance is achieved by YOLOv3 and YOLOv4 models. 

The YOLOv5n achieves an average value of 0.87, 0.878, and 0.895 for precision, recall, and mAP, respectively, which is the lowest in all YOLOv5 versions. The YOLOv5s achieves 0.906% precision, 0.835% recall, and 0.901% mAP for pests detection while the YOLOv5m obtains 0.936% precision, 0.845% recall, and 0.907% mAP. The YOLOv5l and YOLOv5x achieve good results in terms of precision, recall, and mAP as presented in Table 2, where the YOLOv5x outperformes the YOLOv5l by achieving a higher value for precision, recall, and mAP. In the Table 2, it can be observed that the proposed model achieves higher performance than other versions of YOLOv5, which surpasses the YOLOv5x by obtaining higher precision, recall, and mAP values. 

### 5.4. Model Complexity Analysis

The detailed feasibility analysis of the proposed model in terms of parameters, model size, and frame per second (FPS) using CPU is described in Table 3. To approximate the inferencing time, we determine the Giga Floating Point Operations per Second (GFLOPs), model size, and Frame Per Second (FPS) of each model and compare it with the proposed model as presented in Table 3. The higher GFLOPs, model size, and lower inferencing speed are associated with the YOLOV5x, which restricts the system from real-world applications. In Table 3, it can be seen that the proposed model is a suitable choice for pest detection due to the higher inference speed and lower GFLOPs and model size, which increase the potential of our model to be implemented in real-time. 

### 5.5. Visual Result of the Proposed Model

**Figure 8:**
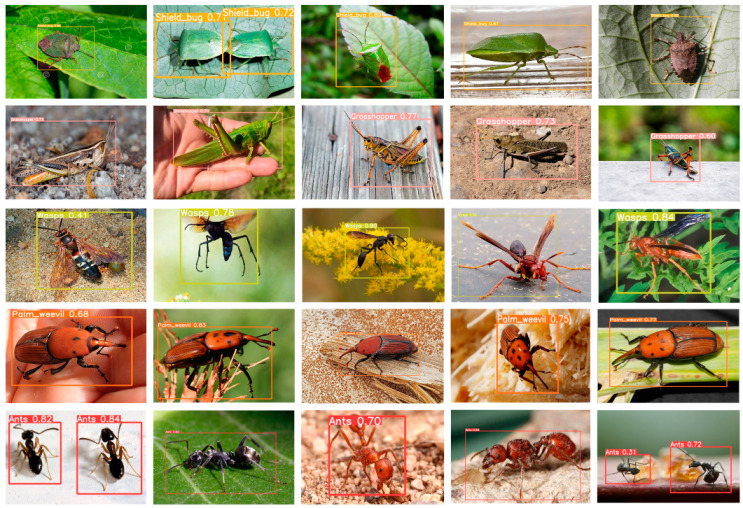
Represents the visual results of the proposed model which show the model effective analysis.

The proposed model is utilized to detect five kinds of pests in the natural environment and check the classification and identification performance of the model. The visual results of our model are shown in Figure 8. In this figure, the proposed model achieves better performance in terms of detection and classification, where the proposed model draws an accurate bounding box around the object and assigns a correct class label to the object. Thus, the visualized results of our model show real-time applicability.

## 6. Conclusions

In this research, we have experimented with nine different object detection models including the proposed model, which is the most efficient and accurate model as proven by the experimental section. All the experiments were performed using the manually collected dataset, which consists of five different classes i.e., ants, grasshopper, palm weevils, shield bugs, and wasps. Our model surpassed the state-of-the-art models by achieving higher values for precision, recall, and mAP, i.e., 0.018, 0.015, and 0.011, respectively. Furthermore, the proposed models can be deployed in real-time due to the higher inferencing speed with lower GFLOPs calculations and model size. As a result, the proposed model is highly capable of detecting and recognizing different types of species in real time. 

In the future, we aim to increase the model performance with reduced model size and GFLOPs calculations by introducing novel mechanisms in the backbone architecture and using vision transformer for pests detection. Further, we plan to increase the number of training and pest species in the dataset to increase the model’s robustness.

## Figures and Tables

**Table 1:** Tabulated form of the self-created dataset.

Class	Number of Images
Ants	392
Grasshopper	315
Palm_weevil	148
Shield_bug	392
Wasps	318

**Table 2:** Comparative analysis of the proposed model with various versions of the YOLOv5 models.

Models	Classes	Precision	Recall	mAP
Faster RCNN	All	0.92	0.89	0.924
	Ants	0.73	0.74	0.76
	Grasshopper	0.98	0.99	1
	Palm_weevil	0.99	0.88	0.98
	Shield_bug	0.96	0.97	1
	Wasps	0.94	0.86	0.91
YoloV3	All	0.82	0.87	0.86
	Ants	0.59	0.75	0.64
	Grasshopper	0.86	0.84	0.91
	Palm_weevil	0.91	0.93	0.95
	Shield_bug	0.88	0.91	0.93
	Wasps	0.87	0.9	0.88
YoloV4	All	0.85	0.87	0.89
	Ants	0.65	0.76	0.71
	Grasshopper	0.87	0.83	0.93
	Palm_weevil	0.93	0.92	0.96
	Shield_bug	0.9	0.94	0.95
	Wasps	0.88	0.91	0.89
Yolov5n	All	0.87	0.878	0.895
	Ants	0.573	0.74	0.677
	Grasshopper	0.923	0.875	0.944
	Palm_weevil	1	0.983	0.995
	Shield_bug	0.933	0.972	0.978
	Wasps	0.922	0.821	0.881
Yolov5s	All	0.906	0.835	0.901
	Ants	0.797	0.679	0.781
	Grasshopper	0.904	0.875	0.88
	Palm_weevil	9.965	1	0.995
	Shield_bug	0.973	0.915	0.977
	Wasps	0.888	0.706	0.871
YoloV5m	All	0.936	0.845	0.907
	Ants	0.861	0.655	0.731
	Grasshopper	0.948	1	0.995
	Palm_weevil	1	0.816	0.995
	Shield_bug	0.943	0.934	0.97
	Wasps	0.93	0.821	0.846
Yolov5l	All	0.849	0.89	0.917
	Ants	0.723	0.721	9.756
	Grasshopper	1	0.933	0.995
	Palm_weevil	0.758	1	0.984
	Shield_bug	0.885	0.958	0.965
	Wasps	0.881	0.839	0.886
Yolo5x	All	0.912	0.882	0.921
	Ants	0.697	0.724	0.764
	Grasshopper	0.969	1	0.995
	Palm_weevil	1	0.866	0.975
	Shield_bug	0.972	0.978	0.992
	Wasps	0.922	0.841	0.89
Our model	All	0.938	0.896	0.934
	Ants	0.79	0.76	0.80
	Grasshopper	0.98	1	0.996
	Palm_weevil	1	0.886	0.98
	Shield_bug	0.975	0.978	0.993
	Wasps	0.947	0.856	0.9

**Table 3:** Comparing the model complexity analysis of the proposed model with five different versions of YOLOv5.

Model	GFLOPs	Model size	FPS (CPU)
YOLOV5n	4.2	3.65	31.02
YOLOV5s	16	14.1	21.25
YOLOV5m	48.3	40.2	10.16
YOLOV5l	108.3	88.5	6.62
YOLOV5x	204.7	165	3.90
The proposed model	4.8	13	28.60
